# Characterization of maturation of neuronal voltage-gated sodium channels SCN1A and SCN8A in rat myocardium

**DOI:** 10.1186/s40348-015-0015-5

**Published:** 2015-03-11

**Authors:** Ulrich Krause, Christian Alflen, Michael Steinmetz, Matthias J Müller, Thomas Quentin, Thomas Paul

**Affiliations:** Department of Pediatric Cardiology and Intensive Care Medicine, University Medical Center, Georg August University, Göttingen, Robert-Koch-Str. 40, 37099 Göttingen, Germany

**Keywords:** Voltage-gated sodium channel, SCN1A, SCN5A, SCN8A, Developmental expression, Myocardium, Rat

## Abstract

**Background:**

Sodium channels predominantly expressed in brain are expressed in myocardial tissue and play an important role in cardiac physiology. Alterations of sodium channels are known to result in neurological disease in infancy and childhood. It will be of interest to study the expression of brain-type sodium channels in the developing myocardium.

**Methods:**

The expression of neuronal sodium channels (SCN1A, SCN8A) and the cardiac isoform SCN5A in the developing rat myocardium was studied by rtPCR, Western blot, and immunohistochemistry at different stages of antenatal and postnatal development.

**Results:**

Significant changes of sodium channel expression during development were detected. Whereas SCN5A RNA increased to maximum levels on day 21 after birth, the highest SCN1A RNA levels were detected on day 1 to 7 after birth. SCN8A RNA was maximally expressed during embryonic development. At the protein level, the amount of SCN5A protein increased along with the RNA level. SCN1A protein level decreased after birth in contrast to RNA expression. Western blot could not detect SCN8A protein in the myocardium at any stage of development. Immunohistochemistry however proved the presence of SCN8A protein in the developing rat myocardium.

**Conclusions:**

Heart- and brain-type sodium channels are differentially expressed during ontogenesis. The high expression level of SCN1A in the perinatal period and early infancy indicates its importance in preserving a regular cardiac rhythm in this early phase of life. Altered regulation of sodium channels might result in severe cardiac rhythm disturbances.

## Background

Voltage-gated sodium channels (VGSCs) conduct the sodium current, responsible for the rapid initial upstroke of the action potential in excitable cells [1,2]. To date, ten different isoforms of the pore-forming VGSC α-subunit have been described in the literature [3,4]. The quantitative expression of each isoform varies between different tissues [3]. SCN5A is the VGSC mainly expressed in the heart. Besides cardiac-specific pore-forming VGSC α-subunits, there is evidence that other forms of VGSC α-subunits, which are mainly expressed in skeletal muscle and neuronal tissue, are present in cardiac tissue [5-9]. Recently, the functional relevance of myocardial expression of other subtypes of VGSC, which otherwise are predominantly expressed in neuronal tissue, has been shown [5,6]. Brain-type VGSC seems to play an important role in excitation contraction coupling as well as in normal sinoatrial node function [10,11]. To date, little is known about the differential expression of voltage-gated sodium channel gens in the developing myocardium. In neuronal tissue, the differential expression of voltage-gated sodium channels during ontogenesis has been shown recently [12]. As sodium channels play an important role in normal brain development, it is reasonable to assume that the same is true for normal myocardial development [13]. Recent data acquired from whole cell patch clamp experiments using human cardiac myocytes from pediatric as well as adult patients suggest developmental changes in sodium current properties of cardiac myocytes [14]. However, data on the details of the developmental expression of VGSC and its regulation in the myocardium are rare yet.

The aim of the present study was to elucidate the differential expression of heart-type as well as brain-type VGSC α-subunits during ontogenesis in the rat myocardium on RNA and protein level in a quantitative approach. As brain-type VGSC seem to play a crucial role for cardiac rhythmogenesis within the sinuatrial node [11] and may contribute to the etiology of sudden cardiac death in patients suffering from Dravet Syndrome, it was of interest whether any of the analyzed VGSC genes shows predominant expression either within the ventricular or the atrial myocardium.

## Methods

### Tissue samples

Cardiac and brain tissue was obtained from Wistar rats (five animals/age group) of either gender raised according to the guidelines for the care and use of laboratory animals by the US National Institute of Health. The local institutional Animal Care Committee (Georg August University, Göttingen) had approved all procedures. The animals were sacrificed by either decapitation (age 1 and 7 days) or cervical dislocation (age 21 days and 6 weeks) according to the local institutional animal care guidelines. Whole hearts and brains were taken out quickly and frozen in liquid nitrogen (Universal Industrial Gases, Easton, PA, USA) for RNA extraction and for Western blot analysis. At the developmental stages of 21 days and 6 weeks, atrial myocardium was separated from the ventricles before further processing. For preparation of embryonic tissue, pregnant rats were sacrificed by cervical dislocation on day 17 of gestation. Embryos were taken out of the uterus, and whole hearts and brains were quickly excised and frozen in liquid nitrogen for further procession.

For immunohistochemistry, whole hearts and brains were fixated in formaldehyde (Merck & Co., Inc., Whitehouse Station, NJ, USA) for 24 h. For immunohistochemistry from embryonic tissue, whole embryos were fixated in formaldehyde for 24 h, respectively. After initial fixation, tissue was divided into two halves according to the desired direction of slicing and fixated with formaldehyde for another 24 h.

Following fixation, tissue was embedded in paraffin (Merck & Co., Inc., Whitehouse Station, NJ, USA) and subsequently slices of 3-μm thickness were obtained using a microtome (RM 2165, Leica Microsystems GmbH, Wetzlar, Germany). The final slices were then further processed for immunohistochemistry.

### RNA purification

After frozen tissue was pulverized in a mortar cooled in liquid nitrogen, RNA extraction was accomplished using the peqGOLD TriFast™ reagent (PEQLAB Biotechnologie GmbH, Erlangen, Germany). For RNA purification, the RNeasy Mini Kit™ (Qiagen, Hilden, Germany) was used. Extraction and purification of RNA were conducted according to the manufacturer’s protocol. Purified RNA samples were stored at −80°C until further processing.

### RT-PCR and mRNA quantification

One microgram of RNA was transcribed into cDNA using random hexamere primers and SuperScript® II Reverse Transcriptase (both Invitrogen™, Life Technologies Corporation, Darmstadt, Germany) according to the manufacturer’s instruction using a thermocycler (iCycler®, Bio-Rad Laboratories GmbH, München, Germany). mRNA expression was determined using SYBR®-green on a TaqMan® sequence detector (Applied Biosystems®, Life Technologies Corporation, Darmstadt, Germany). For each gene, intron-spanning primer pairs were used in a final concentration of 0.2 μM (sodium channel genes: Biomers.net GmbH, Ulm, Germany; adiponectin receptor 1 gene: Carl Roth GmbH, Karlsruhe, Germany). Table 1 gives detailed information on the primers used. TaqMan® cycling conditions were as follows: initially, taq-polymerase was activated at 95°C for 15 min. Thereafter, amplification cycle conditions were 30 s at 94°C, 30 s at 58°C, and finally 30 s at 72°C. Amplification cycles were repeated for 40 times. After each run, a melting curve analysis was performed to ensure amplification of correct products. Sodium channel mRNA expression was normalized for levels of the cardiac adiponectin receptor 1 as a housekeeping gene, which is stably expressed in cardiac tissue at all developmental stages [15].

**Table 1:** **Primers used**

**Gene**	**Forward primer**	**Reverse primer**
SCN1A	5′ CCC ACC ACT CAG AAT CTC ATA C 3′	5′ GGC TAT ACA TTG AAC GTC ATC C 3′
SCN5A	5′ AGC CAC CAG TGA TAA CCT C 3′	5′ CTG CAT AAG GTT CGT CAG G 3′
SCN8A	5′ AAC TTC CGA ATC TCA CGG ATG 3′	5′ GTG TGG AAC ATG CAG TAA CCG 3′
AR 1^†^	5′ ATC CTG GTC ACA ATG GGA TAC C 3′	5′ CCT ACG CTG AAT GCT GAG TGA T 3′

Sequences of primers used for rtPCR. ^†^Adiponectin receptor 1.

### Membrane extraction

For membrane extraction, fresh tissue samples were used according to the manufacturer’s protocol (Plasma Membrane Protein Extraction Kit, BioVision, Mountain View, USA).

### Protein purification, SDS-PAGE, and Western blot

Frozen tissue was pulverized in a mortar as described above. After suspending the powder in lysis buffer (20 mM TRIS, 50 mM NaCl, 50 mM NaF, 4 mM Na-pyrophosphate, 0.25 M sucrose, pH 7.4 ad 500 ml ddH_2_O, 10 mM DTT, 1% TritonX-100, protease inhibitors and phosphatase inhibitors; Sigma-Aldrich, Deisenhofen, Germany), protein was precipitated by adding isopropanol to the organic phase of the suspension. After incubation at room temperature for 10 min, the precipitated proteins were sedimented by centrifugation (10,000 × *g*, 10 min, 4°C). The supernatant was discarded and the protein pellet was washed three times with ethanol containing 0.3 mM guanidine hydrochloride (20-min incubation at room temperature, 20-min centrifugation at 10,000 × *g*, 4°C). After a further washing step with ethanol, the protein pellet was dried in a vacuum centrifuge. The protein pellet was dissolved in 1% SDS containing protease inhibitors and phosphatase inhibitors (Sigma-Aldrich, Deisenhofen, Germany). Final protein concentration was determined using a commercially available kit (BCA Protein Assay Kit, Thermo Fisher Scientific Inc., Waltham, MA, USA) according to the manufacturer’s instructions.

A total of 30 to 120 μg cellular protein extract (glyceraldehyde 3-phosphate dehydrogenase (GAPDH) 30 μg, SCN5A 50 μg, SCN1A and SCN8A 120 μg) per lane were subjected to SDS-PAGE (14% polyacrylamide gel and 4 % polyacrylamide gel, respectively). Gels were incubated for 30 min at 37°C. Subsequently, proteins were transferred to a nitrocellulose membrane and the membrane was blocked in Tris-buffered saline (TBS) buffer pH 7.6 containing 5% milk powder for 2 h at room temperature. Incubation with the primary antibody was performed overnight at 4°C in TBS buffer containing 1% Tween and 5% milk powder. Primary antibody dilutions were polyclonal rabbit anti-SCN1A, anti-SCN5A, anti-SCN8A 1:200 (ASC-001, ASC-005, and ASC-009, respectively; all AlomoneLabs, Jerusalem, Israel), and monoclonal rabbit anti-GAPDH 1:2,000 (#2118, Cell Signaling Technology, Danvers, USA). HRP-conjugated secondary antibodies (goat anti-rabbit Ig, BD Pharmingen, San Diego, USA) were used in TBS buffer containing 1% Tween and 5% milk powder for 2 h at room temperature. The following secondary antibody dilutions were used: SCN1A, SCN5A, SCN8A 1:2,000, and GAPDH 1:10,000. Chemiluminescence was detected using Super Signal West Femto reagent (Thermo Fisher Scientific Inc., Waltham, USA) and a LAS 4000 mini imaging system (GE Healthcare, Buckinghamshire, UK).

In order to evaluate the amount of proteins, the optical density of the respective protein representing bands was evaluated and normalized to the optical density of the bands representing the housekeeping protein (Bio Photometer®, Eppendorf AG, Hamburg, Germany).

### Immunohistochemistry

Tissues slices for immunohistochemistry were prepared as described above. Paraffin slices were subsequently incubated three times in xylol for 8 min before rehydration was accomplished by rinsing with ethanol of decreasing concentration (100%, 96%, 70%, 50%, and 30% for 8 min, respectively). This step was followed by 8 min rinsing with deionized water (all chemicals by Sigma-Aldrich, Deisenhofen, Germany). Rinsing was followed by 20-min incubation at 90°C. Following this procedure, tissue samples were washed 5 times with Tris-buffered saline and Tween 20 (TTBS) and treated with DAKO peroxidase blocking reagent for 17 min thereafter (RMR 662, Biocare, Concord, CA, USA). Subsequently, they were rinsed with TTBS for 5 min again. After incubation with Rodent Block R blocking reagent (Biocare, Concord, CA, USA) and rinsing with TTBS, primary antibodies and substitution controls were applied respectively and incubated overnight. Antibody concentrations were 1:200 for SCN1A, 1:400 for SCN5A, and 1:600 for SCN8A. The secondary antibody (Rabbit on Rodent, Biocare, Concord, CA, USA) was preincubated for 30 min with XR Factor (Biocare, Concord, CA, USA) in a concentration of 1:76 in order to minimize unspecific staining. After rinsing with TTBS and deionized water, counterstaining with hemalaun was performed. Finally, water was extracted by incubation in 96% and 100% ethanol and xylol for 1, 2, and 5 min, respectively.

### Statistical evaluation

Results are expressed as mean percent change of mRNA expression or protein amount ± standard deviation (SD) compared to developmental stage E17 set at 100%. Statistical analysis was performed using one-way *analysis of variance* (ANOVA). Significance of differences was established using a Tukey’s test for *post hoc* comparisons. All statistical analysis was performed using GraphPad Prism version 4.00 software (GraphPad Software Inc., La Jolla, CA, USA). *p* values <0.05 were considered to be significant, and *p* values <0.001 were assumed to be highly significant.

## Results

### Differential mRNA expression of VGSC during development

**Figure 1:**
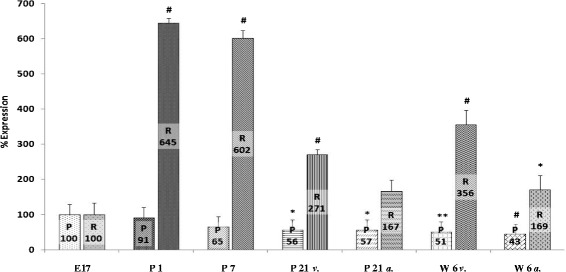
**SCN1A mRNA expression.** Expression of SCN1A mRNA increased dramatically after birth and declined significantly with postnatal development. At the age 21 days and 6 weeks, respectively, mRNA expression in the ventricular myocardium exceeded atrial expression significantly (*p < 0.05; **p < 0.01; ^#^ *p* < 0.001). E17, embryonic day 17 after gestation; P1, 1st postnatal day; P7, 7th postnatal day, P21*v*., 21st postnatal day, ventricular myocardium; P21*a*., 21st postnatal day, atrial myocardium; W6*v*., 6th postnatal week, ventricular myocardium; W6*a*., 6th postnatal week, atrial myocardium; P, protein level; R, mRNA level.

Analysis of mRNA expression of VGSC in the developing rat myocardium revealed differential expression of SCN1A, SCN5A, and SCN8A. A more than sixfold increase of SCN1A mRNA expression was found immediately after birth (645 ± 13%, *n* = 5, *p* < 0.001). As describes above, RNA expression at stage E17 was set to 100% as a reference value for all genes analyzed at any developmental stage. Whereas there was no significant change in the amount of SCN1A mRNA until day 7 of life (602 ± 22%, *n* = 5), a significant drop of mRNA levels was observed on day 21 (241 ± 14%, *n* = 5, *p* < 0.001, ventricular myocardium; 167 ± 32%, *n* = 5, *p* < 0.05, atrial myocardium, respectively) and at the age of 6 weeks (356 ± 40%, *n* = 5, *p* < 0.001, ventricular myocardium; 169 ± 43%, *n* = 5, *p* < 0.05, atrial myocardium, respectively). Compared to prenatal mRNA levels, however, SCNA1 expression was still 1.5 to 3 times higher on day 21 and 6 weeks after birth (Figure 1).

**Figure 2:**
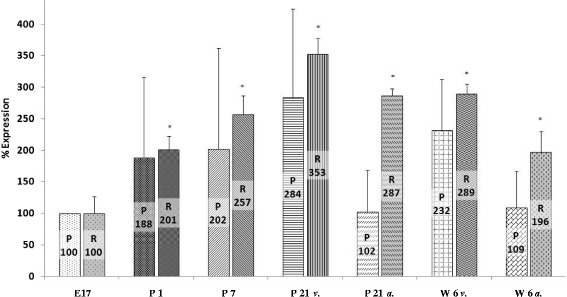
**SCN5A mRNA expression.** Expression of SCN5A mRNA steadily increased after birth compared to the embryonic level of development. The slight drop of mRNA expression after day 21 was not significant. Significance refers to the amount of mRNA on day 17 of embryonic development (Abbreviations and levels of significance as depicted in Figure 1).

Analyzing mRNA the expression of SCN5A at different stages of ontogenesis, the amount of SCN5A mRNA significantly increased after birth to 201 ± 21% on day 1 (*n* = 5, *p* < 0.001), to 257 ± 30% on day 7 (*n* = 5, *p* < 0.001), and to 353 ± 25% on day 21 (*n* = 5, *p* < 0.001), respectively. This observation was similar to the results for SCN1A mRNA in this study. A slight and non-significant drop to 289 ± 16% was found at the age of 6 weeks. However, this amount of SCN5A mRNA was still significantly higher in comparison with the prenatal mRNA amount at stage E17 (*n* = 5, *p* < 0.001, Figure 2).

**Figure 3:**
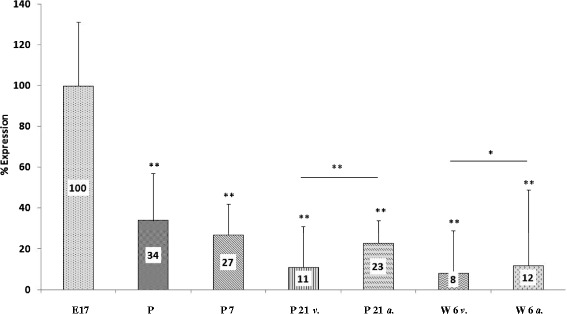
**SCN8A mRNA expression.** Highest expression of SCN8A mRNA was observed on day 17 of gestation and progressively declined after birth. Lowest levels of SCN8A mRNA were measured at age 6 weeks (Abbreviations and levels of significance as depicted in Figure 1).

In contrast to the aforementioned SCN1A and SCN5A genes, mRNA expression of the second brain-type VGSC SCN8A was highest at the embryonic stage of development and consecutively dropped after birth to 34 ± 23% on day 1 of life, to 27 ± 15% on day 7 after birth, to 11 ± 20% on day 21, and finally reached a level of 8 ± 21% in the ventricular myocardium of 6-week-old animals (Figure 3), respectively.

### Differences of mRNA expression in ventricular vs. atrial myocardium

In order to analyze the regional expression of VGSC mRNA in the developing heart, we compared mRNA expression in the ventricular myocardium with mRNA expression in the atrial myocardium at the developmental stages day 21 and 6 weeks. SCN1A and SCN5A mRNA expression was lower in the atria compared to ventricular myocardium (167 ± 32% atrial myocardium vs. 271 ± 14% ventricular myocardium of SCN1A at day 21, *p* < 0.05, *n* = 4; 169 ± 43% atrial myocardium vs. 356 ± 40% ventricular myocardium of SCN1A after 6 weeks, *p* < 0.01, *n* = 5; 287 ± 11% atrial myocardium vs. 353 ± 25% ventricular myocardium of SCN5A at day 21, statistically not significant; 196 ± 34% atrial myocardium vs. 289 ± 16% ventricular myocardium of SCN5A after 6 weeks, statistically not significant) whereas a higher expression of SCN8A mRNA was found at the atrial site (23 ± 11% atrial myocardium vs. 11 ± 20% ventricular, myocardium at day 21, *p* < 0.001, *n* = 5; 12 ± 37% atrial myocardium vs. 8 ± 21% ventricular myocardium after 6 weeks, *p* < 0.001, *n* = 5; Figures 1, 2, and 3, Table 2).

**Table 2:** **SCN1A, SCN5A, and SCN8A expression**

	**P21**			**W6**		
**Gene**	**Atrium**	**Ventricle**	***p***	**Atrium**	**Ventricle**	***p***
SCN1A	167 ± 32%	271 ± 14%*	< 0.05	169 ± 43%	356 ± 40%*	<0.01
SCN5A	287 ± 11%	353 ± 25%	*n.s*.	196 ± 34%	289 ± 16%	*n.s*.
SCN8A	23 ± 11%	11 ± 20%*	< 0.001	12 ± 37%	8 ± 21%*	<0.001

Differential expression of VGSC within the atrial as well as the ventricular myocardium at postnatal developmental stages of 21 days (P21) and 6 weeks (W6). Significant differences are marked by an asterisk (*).

### Differential expression of VGSC protein in the developing rat heart

**Figure 4:**
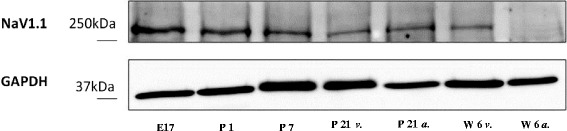
**Analysis of SCN1A protein expression.** In contrast to rtPCR results, Western blot analysis of SCN1A protein expression showed a progressive decline with postnatal development (*upper panel*). As a control, protein expression of GAPDH showed no changes with development (*lower panel*; abbreviations as depicted in Figure 1).

In order to determine the levels of SCN1A, SCN5A, and SCN8A protein (Na_v_1.1, Na_v_1.5, and Na_v_1.6, respectively), Western blot analysis was performed from samples of rat myocardium at the same developmental stages as PCR analysis had been conducted. In contrast to distribution of mRNA expression of SCN1A throughout ontogenetic development, SCN1A protein levels were highest during the embryonic stage of development and initially decreased slightly at day 1 of life (91 ± 47%). On day 7, a further decrease of protein amount of SCN1A was observed (65 ± 43%). Both changes, however, did not reach statistical significance compared to developmental stage E17 (*n* = 3). After 21 days and 6 weeks of life, SCN1A protein amount dropped significantly to 56 ± 38% (ventricular myocardium, day 21), to 57 ± 39% (atrial myocardium, day 21, *p* < 0.05, *n* = 3, ventricular and atrial myocardium), to 51 ± 35% (ventricular myocardium 6 weeks, *p* < 0.01, *n* = 3), and to 43 ± 29% (atrial myocardium 6 weeks, *p* > 0.001, *n* = 3, Figures 1 and 4).

Whereas the protein amount and mRNA expression of SCN1A differed profoundly during development, SCNA5A protein amount almost paralleled mRNA expression. On day 1 and day 7 of postnatal life, SCN5A protein amount increased to 188 ± 127% and 202 ± 160% compared to developmental stage E17. On day 21 and after 6 weeks, a further increase in protein amount of ventricular myocardium was observed (284 ± 140% and 232 ± 81%, respectively). Lower levels of SCN5A protein were detected in atrial myocardium after 21 days and after 6 weeks of life (102 ± 66% and 109 ± 58% respectively). Developmental differences of SCN5A protein amount at various developmental stages as detected however failed to reach statistical significance (*n* = 3, Figure 2).

**Figure 5:**
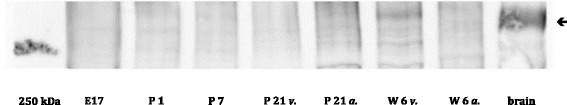
**Analysis of SCN8A protein expression.** SCN8A protein could not be found all in cardiac tissue of any developmental stage. However, a typical band slightly above 250 kDa was found in a Western blot from brain tissue of 6-week-old animals (*arrow*; abbreviations as depicted in Figure 1).

Whereas mRNA of SCN8A was readily detectable at all developmental stages, Western blot analysis from cardiac tissue failed to detect SCN8A protein at all. In rat brain tissue, however, SCN8A protein (NaV 1.6) was easily detectable using an identical Western blot protocol (Figure 5).

### Immunohistochemistry

**Figure 6:**
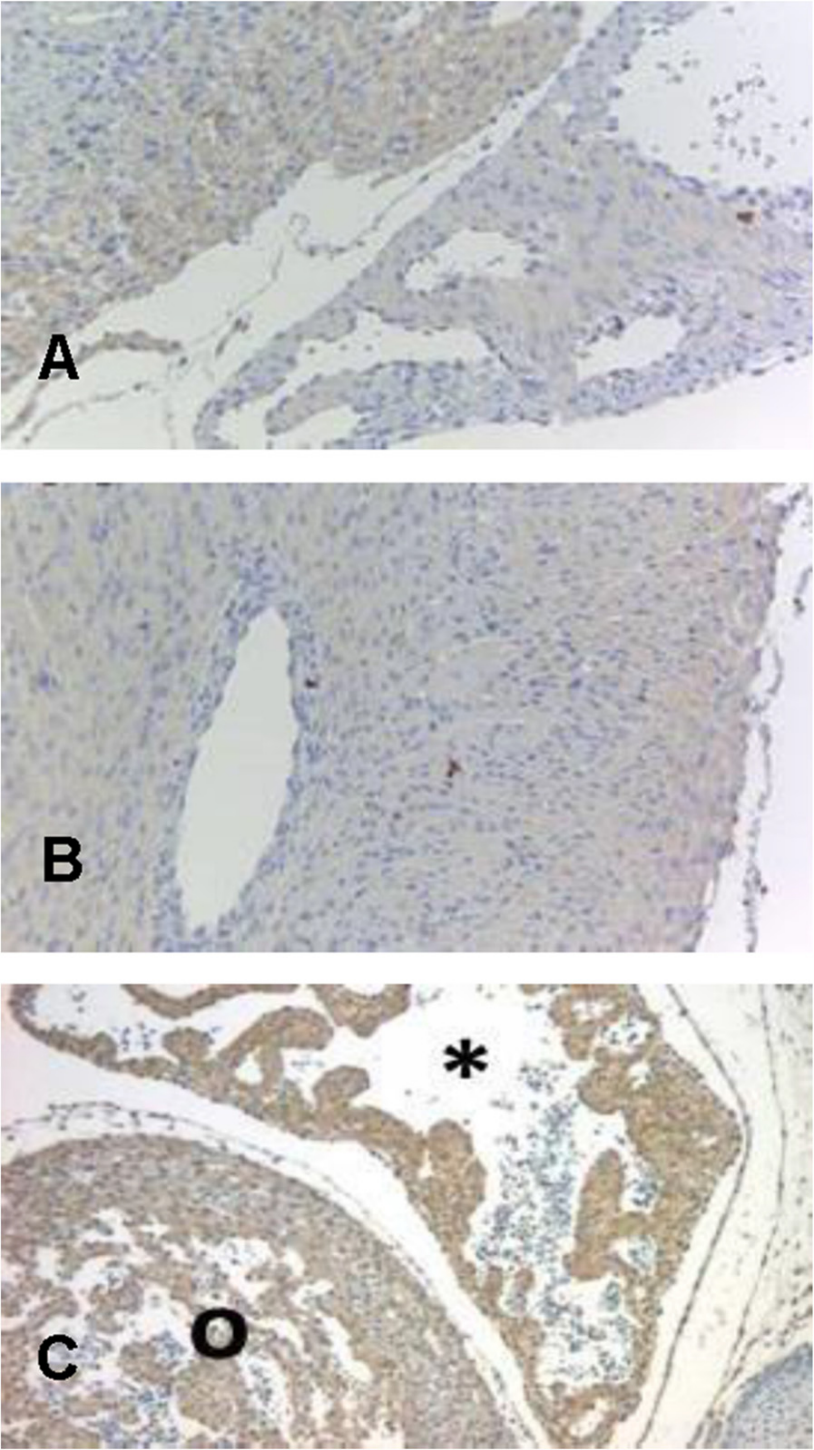
**SCN8A specific immunohistochemistry from tissue samples of different developmental stages (A = P1, B = P7, C = W6).** At developmental stage W6, staining of the atrial myocardium (*asterisk*) was more intense compared with ventricular myocardium (o).

In order to verify the presence of SCN8A protein in the developing rat myocardium, we used an immunohistochemical approach to detect SCN8A protein in cardiac tissue. As depicted in Figure 6, SCN8A protein was detectable at the developmental stages P1, P7, and W6 postnatally. In tissue samples from 6-week-old animals, we found a quite strong signal in the atrial myocardium compared with ventricular myocardium, suggesting a predominantly atrial expression of SCN8A protein at this stage of development.

## Discussion

In previous studies, presence of other than the “cardiac-specific” VGSC had been suspected by the fact that tetrodotoxin (TTX) had been shown to impair left ventricular function. VGSC isoforms preferentially expressed in central nervous tissue are inhibited by nanomolar concentrations of TTX, whereas the cardiac isoform SCN5A is inhibited only by micromolar concentrations of TTX and is therefore considered to be TTX-resistant [4]. In the present study, we were able to prove the expression of brain-type VGSC in the developing rat myocardium. The expression of the cardiac VGSC SCN5A and brain-type VGSC SCN1A and SCN8A was regulated differentially in the developing rat myocardium. Differential expression was demonstrated on RNA level as well as on protein level.

The differential expression of VGSC genes in cardiomyocytes has been shown in a recent study [13]. However, this study did not quantitatively measure the expression of VGSC α-subunit protein but was restricted to quantitative PCR and immunocytochemistry, a non-quantitative method. It is well known that posttranscriptional as well as posttranslational modification regulates the amount of functional VGSC protein in the plasma membrane of excitable cells. Mechanisms like microRNA and protein phosphorylation as well as dephosphorylation probably play a crucial role in such processes [16-19].

Focusing on the time course of VGSC expression in different tissues, it is an interesting finding that in the central nervous system, the differential expression of SCN1A protein has been described to follow a distinct time course. The expression of SCN1A protein in brain tissue was found to steadily increase from birth until the age of 7 months. However, the time course of differential expression in central nervous tissue differs from what we found in myocardial tissue [12]. This obviously might be a result of different laboratory agents and primers used in the present and in the quoted study. However, different demands of cardiomyocytes vs. central nervous neurons should also be taken into consideration to explain the observed differences. The different results may therefore be explained by either different posttranslational modification of SCN1A protein in brain tissue or blockade of SCN1A translation by microRNA. Future studies on pre- and posttranslational processing of SCN1A are required to elucidate the underlying mechanisms of this phenomenon. It is well known that adrenergic stimulation of the heart influences not only gating kinetics of myocardial VGSC but also the amount of VGSC and their distribution within the plasma membrane of the cardiomyocytes [20]. Since a differential developmental expression has been shown for adrenergic receptors in the developing heart as well [21-23], a role of changing adrenergic stimulation in the regulation of myocardial VGSC expression may be anticipated and needs further investigation.

A loss of function mutation of SCN1A has been linked to an epilepsy disorder called Dravet Syndrome (DS). This disease usually manifests within the first year of life and patients with DS are prone to develop sudden cardiac death [24-26]. The differential SCN1A expression in rat myocardium might influence the susceptibility for sudden cardiac death in these children at different ages, since higher incidence of sudden death at young ages in patients with epilepsy has been described in multiple studies [27-30]. In order to further investigate the role of SCN1A in sudden death in infants and children and to elucidate the role of cardiac SCN1A in this setting, it would be worthwhile to study the electrical properties of an SCN1A knockout animal. Such animals have been established [31] but characterization of the electrical properties of the animal cardiomyocytes is still lacking. As SCN1A is highly expressed within the SA node in mice and therefore might play an important role in normal rhythmogenesis [11], a higher amount of SCN1A gene product at earlier developmental stages has been suggested to be responsible for the physiologically higher heart rate at younger age [13].

In order to address the question whether other than cardiac-specific TTX-resistant VGSC are expressed in myocardial tissue, previous work suggested the absence of TTX- and saxitoxin (STX)-sensitive receptors in fetal and neonatal myocardium. The appearance of high-affinity STX and TTX receptors has been shown at later stages of development in cardiac tissue [5,32]. Our results showed a drop of SCN1A as well as SCN8A gene expression after birth, which is in contrast to the results of the studies mentioned before since SCN1A and SCN8A represent TTX-sensitive VGSCs. This may be explained by the fact that skeletal muscle VGSC conduct a high proportion of TTX-sensitive sodium current in cardiomyocytes, and previous studies did not differentiate between VGSC isoforms [5,13,32]. The differential expression of skeletal muscle VGSC however was not analyzed in the present study.

The expression of SCN8A RNA was highest at the embryonic stage of development and decreased progressively after birth. These results are in accordance with the observation of Haufe et al. [13] demonstrating a similar developmental SCN8A RNA expression pattern in mouse myocardium. In the present study, however, we were not able to detect SCN8A protein by means of Western blot analysis. This finding may be due to very small amounts of protein in cardiomyocytes. In order to rule out a methodical error resulting in failure of SCN8A protein detection in the developing rat myocardium by Western blot, we were able to quantify the expression of SCN8A protein from brain samples with an identical experimental setup (Figure 5). In order to prove expression of SCN8A protein in cardiomyocytes, we were able to detect SCN8A protein in rat cardiomyocytes of different developmental stages by means of immunocytochemistry. Quantification of SCN8A protein expression was therefore not possible. At a later developmental stage, we found a more intense labeling for SCN8A protein in the atrial than in the ventricular myocardium, indicating an important role of SCN8A in atrial sodium current conductance. Assuming that protein expression of SCN8A follows RNA expression as has been shown for SCN5A in the present study, the quantification of differential SCN8A protein expression would be of interest. A higher expression of SCN8A during ante- as well as early postnatal stages of development might indicate an important role of SCN8A ensuring proper action potential propagation throughout atrial cardiomyocytes during early development and a shift to other VGSC at later developmental stages and during adulthood.

The developmental expression of the cardiac-specific VGSC SCN5A mainly followed the expected pattern with an increasing amount of RNA as well as protein with ongoing postnatal development. In contrast to SCN1A, expression of RNA and protein paralleled each other. Since SCN5A is the main VGSC expressed in the myocardium and therefore carries the biggest workload, it seems quite reasonable for the cardiomyocyte not to ‘waste’ RNA or channel protein but to express it as functional channel protein within the cellular membrane.

Besides tissue- and cell-specific regulation of ion channel expression, external factors like hypoxemia or glucose depletion have been shown to influence the expression of cardiac ion channels [33,34]. Therefore, as neither oxygen saturation nor glucose levels of the animals were monitored before they were sacrificed, one cannot entirely rule out such external factors influencing the amount of RNA and protein in the present study, although it seems unlikely.

Likewise, a certain ‘contamination’ of RNA and protein levels of SCN1A and SCN8A by afferent cardiac nerve fibers cannot entirely be ruled out. However, we are quite confident that most of the RNA and protein we found was derived from cardiomyocytes since immunostaining in cardiomyocytes was positive for both proteins at different developmental stages and nerve fibers within the myocardium (PGP 9.5 positive) stained only marginally for VGSC.

## Conclusion

VGSC are differentially expressed in the developing rat myocardium. The present study proved a differential expression not only of RNA but also of VGSC α-subunit protein. Future studies are needed to elucidate the regulatory mechanisms of this phenomenon. Since VGSC ß-subunits are of relevance for the functional properties of VGSC, it will be of interest to study potential expression changes of these regulating ß-subunits in futures studies. Furthermore, not only VGSC but also other ion channels may be expressed differentially throughout development. The ontogenic expression profiles of other cardiac ion channels like potassium and calcium channels need to be studied in the future.

### Limitations

The present study was focused on gaining more information on the quantitative changes of myocardial VGSC expression on RNA level as well as on protein level. However, the informative value of atrial vs. ventricular expression is limited by the fact that RNA and protein levels were measured separately for the atria and the ventricles only at the age of 21 days and 6 weeks and not at earlier developmental stages. It will be the purpose of future studies to elucidate the regulatory mechanisms of this phenomenon. Comparing our data with the results from other studies, an impact of different laboratory agents and different primers cannot be ruled out and may explain at least some of the differences found. In the present study, we did not examine changes of membrane sodium current conducted by the ion channels at different stages of myocardial development. Functional testing, however, will be of interest to understand the impact of differential ion channel expression on myocardial excitation. Therefore, electrophysiological studies of myocardial membrane current properties at different developmental stages may help to improve our understanding. Finally, the present study was conducted on rodents and results may therefore not apply to humans.

## Abbreviations

E17: day 17 of gestation
P1: day 1 of postnatal development
P7: day 7 of postnatal development
P21: day 21 of postnatal development
W6: week 6 of postnatal development
VGSC: voltage-gated sodium channel

## References

[1] Balser JR (2001). The cardiac sodium channel: gating function and molecular pharmacology. J Mol Cell Cardiol.

[2] Catterall WA (1992). Cellular and molecular biology of voltage-gated sodium channels. Physiol Rev.

[3] Catterall WA (2012). Voltage-gated sodium channels at 60: structure, function and pathophysiology. J Physiol.

[4] Goldin AL (2001). Resurgence of sodium channel research. Ann Rev Physiol.

[5] Rogart RB, Cribbs LL, Muglia LK, Kephart DD, Kaiser MW (1989). Molecular cloning of a putative tetrodotoxin-resistant rat heart Na+ channel isoform. Proc Natl Acad Sci U S A.

[6] Sills MN, Xu YC, Baracchini E, Goodman RH, Cooperman SS, Mandel G, Chien KR (1989). Expression of diverse Na+ channel messenger RNAs in rat myocardium. Evidence for a cardiac-specific Na+ channel. J Clin Invest.

[7] Baruscotti M, Westenbroek R, Catterall WA, DiFrancesco D, Robinson RB (1997). The newborn rabbit sino-atrial node expresses a neuronal type I-like Na+ channel. J Physiol.

[8] Huang B, El-Sherif T, Gidh-Jain M, Qin D, El-Sherif N (2001). Alterations of sodium channel kinetics and gene expression in the postinfarction remodeled myocardium. J Cardiovasc Electrophysiol.

[9] Pereon Y, Lande G, Demolombe S, Nguyen The Tich S, Sternberg D, Le Marec H, David A (2003). Paramyotonia congenita with an SCN4A mutation affecting cardiac repolarization. Neurology.

[10] Maier SK, Westenbroek RE, Schenkman KA, Feigl EO, Scheuer T, Catterall WA (2002). An unexpected role for brain-type sodium channels in coupling of cell surface depolarization to contraction in the heart. Proc Natl Acad Sci U S A.

[11] Maier SK, Westenbroek RE, Yamanushi TT, Dobrzynski H, Boyett MR, Catterall WA, Scheuer T (2003). An unexpected requirement for brain-type sodium channels for control of heart rate in the mouse sinoatrial node. Proc Natl Acad Sci U S A.

[12] Wang W, Takashima S, Segawa Y, Itoh M, Shi X, Hwang SK, Nabeshima K, Takeshita M, Hirose S (2011). The developmental changes of Na(v)1.1 and Na(v)1.2 expression in the human hippocampus and temporal lobe. Brain Res.

[13] Haufe V, Camacho JA, Dumaine R, Gunther B, Bollensdorff C, von Banchet GS, Benndorf K, Zimmer T (2005). Expression pattern of neuronal and skeletal muscle voltage-gated Na+ channels in the developing mouse heart. J Physiol.

[14] Cai B, Mu X, Gong D, Jiang S, Li J, Meng Q, Bai Y, Liu Y, Wang X, Tan X, Yang B, Lu Y (2011). Difference of sodium currents between pediatric and adult human atrial myocytes: evidence for developmental changes of sodium channels. Int J Biol Sci.

[15] Steinmetz M, Quentin T, Poppe A, Paul T, Jux C (2005). Changes in expression levels of genes involved in fatty acid metabolism: upregulation of all three members of the PPAR family (alpha, gamma, delta) and the newly described adiponectin receptor 2, but not adiponectin receptor 1 during neonatal cardiac development of the rat. Basic Res Cardiol.

[16] Fabian MR, Sonenberg N, Filipowicz W (2010). Regulation of mRNA translation and stability by microRNAs. Ann Rev Biochem.

[17] Xiao L, Xiao J, Luo X, Lin H, Wang Z, Nattel S (2008). Feedback remodeling of cardiac potassium current expression: a novel potential mechanism for control of repolarization reserve. Circulation.

[18] Yanagita T, Kobayashi H, Yamamoto R, Kataoka H, Yokoo H, Shiraishi S, Minami S, Koono M, Wada A (2000). Protein kinase C-alpha and -epsilon down-regulate cell surface sodium channels via differential mechanisms in adrenal chromaffin cells. J Neurochem.

[19] Baek JH, Rubinstein M, Scheuer T, Trimmer JS (2014). Reciprocal changes in phosphorylation and methylation of mammalian brain sodium channels in response to seizures. J Biol Chem.

[20] Shibata EF, Brown TL, Washburn ZW, Bai J, Revak TJ, Butters CA (2006). Autonomic regulation of voltage-gated cardiac ion channels. J Cardiovasc Electrophysiol.

[21] Rybin VO, Pak E, Alcott S, Steinberg SF (2003). Developmental changes in beta2-adrenergic receptor signaling in ventricular myocytes: the role of Gi proteins and caveolae microdomains. Mol Pharm.

[22] Cao XJ, Li YF (2009). Alteration of messenger RNA and protein levels of cardiac alpha(1)-adrenergic receptor and angiotensin II receptor subtypes during aging in rats. Can J Cardiol.

[23] Wadhawan R, Tseng YT, Stabila J, McGonnigal B, Sarkar S, Padbury J (2003). Regulation of cardiac beta 1-adrenergic receptor transcription during the developmental transition. Am J Physiol Heart Circ Physiol.

[24] Annegers JF, Coan SP (1999). SUDEP: overview of definitions and review of incidence data. Seizure.

[25] Aurlien D, Larsen JP, Gjerstad L, Tauboll E (2012). Increased risk of sudden unexpected death in epilepsy in females using lamotrigine: a nested, case-control study. Epilepsia.

[26] Shorvon S, Tomson T (2011). Sudden unexpected death in epilepsy. Lancet.

[27] Tennis P, Cole TB, Annegers JF, Leestma JE, McNutt M, Rajput A (1995). Cohort study of incidence of sudden unexplained death in persons with seizure disorder treated with antiepileptic drugs in Saskatchewan, Canada. Epilepsia.

[28] Leestma JE, Walczak T, Hughes JR, Kalelkar MB, Teas SS (1989). A prospective study on sudden unexpected death in epilepsy. Ann Neurol.

[29] Nashef L, Fish DR, Garner S, Sander JW, Shorvon SD (1995). Sudden death in epilepsy: a study of incidence in a young cohort with epilepsy and learning difficulty. Epilepsia.

[30] Ficker DM, So EL, Shen WK, Annegers JF, O'Brien PC, Cascino GD, Belau PG (1998). Population-based study of the incidence of sudden unexplained death in epilepsy. Neurology.

[31] Yu FH, Mantegazza M, Westenbroek RE, Robbins CA, Kalume F, Burton KA, Spain WJ, McKnight GS, Scheuer T, Catterall WA (2006). Reduced sodium current in GABAergic interneurons in a mouse model of severe myoclonic epilepsy in infancy. Nat Neursc.

[32] Renaud JF, Kazazoglou T, Lombet A, Chicheportiche R, Jaimovich E, Romey G, Lazdunski M (1983). The Na+ channel in mammalian cardiac cells. Two kinds of tetrodotoxin receptors in rat heart membranes. J Biol Chem.

[33] Peers C (2002). The G. L. Brown Prize Lecture. Hypoxic regulation of ion channel function and expression. Exp Physiol.

[34] Temsah RM, Kawabata K, Chapman D, Dhalla NS (2001). Modulation of cardiac sarcoplasmic reticulum gene expression by lack of oxygen and glucose. FASEB J.

